# Large-Scale Protein and Phosphoprotein Profiling to Explore Potato Resistance Mechanisms to *Spongospora subterranea* Infection

**DOI:** 10.3389/fpls.2022.872901

**Published:** 2022-04-14

**Authors:** Sadegh Balotf, Calum R. Wilson, Robert S. Tegg, David S. Nichols, Richard Wilson

**Affiliations:** ^1^New Town Research Laboratories, Tasmanian Institute of Agriculture, University of Tasmania, New Town, TAS, Australia; ^2^Central Science Laboratory, University of Tasmania, Hobart, TAS, Australia

**Keywords:** proteomics, phosphoproteomics, potato, powdery scab, *Spongospora subterranea*

## Abstract

Potato is one of the most important food crops for human consumption. The soilborne pathogen *Spongospora subterranea* infects potato roots and tubers, resulting in considerable economic losses from diminished tuber yields and quality. A comprehensive understanding of how potato plants respond to *S. subterranea* infection is essential for the development of pathogen-resistant crops. Here, we employed label-free proteomics and phosphoproteomics to quantify systemically expressed protein-level responses to *S. subterranea* root infection in potato foliage of the susceptible and resistant potato cultivars. A total of 2,669 proteins and 1,498 phosphoproteins were quantified in the leaf samples of the different treatment groups. Following statistical analysis of the proteomic data, we identified oxidoreductase activity, electron transfer, and photosynthesis as significant processes that differentially changed upon root infection specifically in the resistant cultivar and not in the susceptible cultivar. The phosphoproteomics results indicated increased activity of signal transduction and defense response functions in the resistant cultivar. In contrast, the majority of increased phosphoproteins in the susceptible cultivar were related to transporter activity and sub-cellular localization. This study provides new insight into the molecular mechanisms and systemic signals involved in potato resistance to *S. subterranea* infection and has identified new roles for protein phosphorylation in the regulation of potato immune response.

## Introduction

Potato (*Solanum tuberosum*), after rice and wheat, is the third most important and valuable staple crop for human consumption globally, and therefore, sustainable production of potato is vital for global food security. Potato production is threatened by plant pathogens, such as fungi, viruses, bacteria, and protozoa (Liu et al., 2016; Wang et al., 2021). *Spongospora subterranea* is a biotrophic soil inhabiting plant pathogen and one of the most economically significant potato diseases. This pathogen invades both roots and tubers resulting in root disfunction, root galling, and powdery scab lesions on tubers in potato (Amponsah et al., 2021). It has been estimated that tuber infection by *S. subterranea* accounts for annual losses of A$13.4M in the Australian processing potato sector alone (Wilson, 2016). Currently, there are no strategies for effective control of *S. subterranea* root and tuber diseases. Resistance to *S. subterranea* infection in potato cultivars has been examined in several studies (Falloon et al., 2003, 2016; Nitzan et al., 2009; Houser and Davidson, 2010; Merz et al., 2012). These studies provided a ranking for relative susceptibility to root galling or powdery scab of tuber. However, there is a lack of detailed knowledge of the molecular basis underlying resistance to *S. subterranea* infection. Thus, an understanding of the regulatory principles underlying *Spongospora*–potato interactions is important.

The availability of genome sequences of potato (Xu et al., 2011) and the draft genome of *S. subterranea* (Ciaghi et al., 2018) makes sequence-based “omics” studies more accessible to potato powdery scab researchers. Proteomics has become a major contributor in investigating plant–pathogen interaction (Balotf et al., 2022) and has led to the discovery of numerous proteins that are expressed during plant–pathogen communication. Although the study of this interaction has frequently focused on transcriptome and proteome levels, the activities of many proteins are further regulated by post-translational modifications (PTMs; Liu et al., 2021). Protein phosphorylation at serine, tyrosine, and threonine residues is a rapid mechanism for controlling cellular processes (Thurston et al., 2005) and is one of the most frequently studied PTMs. Reversible protein phosphorylation is vital for the plant immune signaling in response to pathogen attack (Park et al., 2012). For example, Pang et al. (2020) used label-free proteomics and phosphoproteomics analysis of *Arabidopsis* guard cells in response to a bacterial pathogen. They showed that phosphorylation and dephosphorylation of WRKY transcription factors play a crucial role in regulating plant immunity. In another study in *Arabidopsis*, protein phosphorylation was required to produce reactive oxygen species (ROS) during immunity against virulent necrotrophic fungus (Kadota et al., 2019). Zhang et al. (2019) profiled the phosphoproteome of cotton roots in response to the soilborne plant pathogenic fungus *Verticillium dahliae* infection. The abundance of 30 phosphoproteins common to the susceptible and resistant cotton lines was differentially changed after inoculation with *V. dahliae*. These proteins were correlated with resistance against fungal infection and primarily involved in signal transduction, plant–pathogen interactions, and metabolic processes.

Integration of proteomics with phosphoproteomics can further expand the understanding of molecular events involved in host–pathogen interactions (Thurston et al., 2005). Therefore, in this study, we combined shotgun proteomics with phosphoproteome analysis of leaves from resistant and susceptible potato cultivars in response to *S. subterranea* root infection. This study provides a novel insight for further investigations of biological processes and systemic signals involved in potato defense responses to *S. subterranea*, which holds promise for potential benefits in plant breeding programs.

## Materials and Methods

### Plant Growth and Pathogen Infection

Potato plants obtained from single-node cuttings were grown in a Murashige and Skoog (MS) medium. Around 40 mg/L of ascorbic acid, 30 g/L of sucrose, and 500 mg/L of casein hydrolysate were added to the MS medium. Two potato cultivars that differ in resistance to *S. subterranea* were used; Iwa is a highly susceptible cultivar, and Gladiator, exhibits strong resistance to both tuber and root disease caused by *S. subterranea* (Balendres et al., 2016). *Spongospora subterranea* inoculum was obtained from field-collected powdery scab-infected tubers and semi-purified using a Ludox column centrifugation method (Balotf et al., 2020). Tissue-cultured plantlets (21-day-old) of each cultivar were inoculated by dipping their roots into the inoculum suspension of *S. subterranea* for 1 h. Uninoculated plants of each cultivar were suspended in sterile water only, for 1 h. The seedlings were transplanted into 2 L plastic pots containing sterilized potting mix and were maintained under controlled conditions in the greenhouse. To ensure ongoing disease pressure, an additional 20 ml of fresh inoculum suspension of *S. subterranea* was added to each pot, 2 weeks after planting. Plant growth condition and inoculation has been described in Balotf et al. (2021). After 6 weeks in the greenhouse, the leaves of the infected and control plants were collected, frozen in liquid nitrogen, and stored at −80°C for protein extraction (*n* = 4).

### Chlorophyll Fluorescence

Two days before sampling, the maximal photochemical efficiency of PSII was estimated by measuring the chlorophyll variable (Fv) and maximal (Fm) and fluorescence (Fv/Fm ratio) using Optiscan OS-30p + fluorometer (Opti-Science, Hudson, NH, United States). The measurements were assessed in the leaves adapted to darkness using leaf clips. Five biological replicates were used per treatment for chlorophyll fluorescence measurements.

### Sample Validation Using PCR

PCR was performed with primer pairs targeted to the 18S rRNA gene to confirm the presence of *S. subterranea* in the root of the inoculated plants. The genomic DNA was extracted from 50 mg of each root sample of all plants using the DNeasy Plant Pro kit (Qiagen, Hilden, Germany). The amount of DNA was determined using a Qubit™ dsDNA B.R. Assay Kit (Invitrogen, Waltham, MA). Sequences of oligonucleotides were as follows: F: GTGAACTGCGGAAGGACATT and R: CGTCACCCTTCAACAGACAA. PCR was carried out in 20 μl total reaction volume with the following program: 30 cycles of 94°C for 30 s, 59°C for 30 s, and 72°C for 60 s, followed by 72°C for 5 min. The Ludox-purified *S. subterranea* sporosori was used as a positive control for the PCR.

### Protein Extraction, Digestion, and Phosphopeptide Enrichment

The frozen leaves were homogenized (150 mg leaves in 450 μl extraction buffer) using a Fast Prep-24 bead beater (Mp Biomedicals, Seven Hills, NSW, Australia) for 60 s. The extraction buffer contained 100 mM NaCl, protease, and phosphatase inhibitor cocktails (Roche Diagnostics, NSW, Australia), 1 mM dithiothreitol (DTT), 5 mM EDTA and 50 mM Tris, pH 8.0. Extracts were then centrifuged at 16,000 *g* for 10 min in a cold room (4°C), and the supernatant was collected. Protein concentration was estimated using the Qubit protein assay kit (Invitrogen, Waltham, MA). For each sample, 250 μg proteins were transferred to a new tube and precipitated by adding 9 vol of absolute acetone chilled to −20°C and kept at −20°C overnight. Samples were then centrifuged at 10,000 *g* for 8 min, and the pellet was washed using cold acetone. Protein pellets were resuspended in SDS buffer (5% SDS and 50 mM ammonium bicarbonate). Subsequently, DTT was added to a final concentration of 20 mM, and proteins were reduced for 10 min at 90°C followed by alkylation with 40 mM iodoacetamide for 30 min at room temperature. The samples were then digested using trypsin/LysC (Promega) at a ratio of 50:1 protein:enzyme using S-Trap™ mini columns (Protifi, Farmingdale, NY, United States) according to manufacturer’s instructions. Peptides were desalted using Pierce peptide desalting columns (Thermo Fisher Scientific, Waltham, MA, United States) and the eluted peptides subsampled into separate Lo-bind Eppendorf tubes for total peptide analysis (30  μl) and phosphopeptide enrichment (270  μl) prior to evaporation using a SpeedVac concentrator. MagReSyn Ti-IMAC HP magnetic beads (ReSyn Biosciences, Gauteng, South Africa) were prepared for phosphopeptide binding using three washes in loading buffer [0.1 M glycolic acid in acetonitrile/water/TFA (80:15:5)]. Dried peptide samples were reconstituted in loading buffer then added to the beads, followed by sequential washing and elution of the phosphopeptide-enriched samples as per manufacturer’s instructions.

### LC–MS/MS Analysis and Data Processing

For total peptide analysis, dried peptides were reconstituted in 12 μl of HPLC loading buffer (2% acetonitrile and 0.05% TFA in water) and approximately 1 μg peptides were analyzed by nanoLC-MS/MS using a Q-Exactive HF and Ultimate 3000 nanoHPLC system (Thermo Fisher Scientific, Waltham, MA, United States). Peptides were loaded onto a 20 mm PepMap 100 C18 trapping column (3 mm C18) at 5 μl min^−1^. Using a 2 h segmented gradient from mobile phase A (0.1% formic acid in water) to mobile phase B [0.08% formic acid in acetonitrile/water (80:20)], peptides were separated at 300 nl min^−1^ on a 250 mm PepMap 100 C18 analytical column at 45°C. For phosphopeptide analysis, the dried samples were reconstituted in 10 μl of HPLC loading buffer, of which 4  μl was injected then separated under the same conditions, using a shorter (1 h) segmented gradient. The data-independent acquisition mass spectrometry (DIA-MS) method used for both total peptide and phosphopeptide analysis has been previously described in Balotf et al. (2021). Raw MS files were processed using Spectronaut software (v15) using the directDIA approach. Spectral libraries were generated independently from the total peptide and phosphopeptide samples using the Pulsar search engine to search the *Solanum tuberosum* UniProt reference proteome (UP000011115) comprising 53,106 entries. Default search settings were used, with the exception that for phosphosite identification the PTM localization filter was activated (minimum threshold 0.75) and phospho (S/T/Y) was included as an additional variable modification. Relative quantitation between samples at the proteome or phosphoproteome levels was then achieved by targeted re-extraction of DIA-MS2 spectra from respective total peptide or phosphopeptide libraries. Data were exported from Spectronaut as a PTM site report for the phosphopeptide data and a protein group pivot report for the proteomics data set.

### Statistics and Bioinformatic Analysis

For both proteome and phosphoproteome analysis, the data were imported into Perseus v1.6.14.0 (Tyanova et al., 2016) and log_2_-transformed, followed by imputation of missing values from the normal distribution, according to default Perseus settings. Proteins were classified as significantly altered in abundance between groups on the basis of Student’s *t*-test (FDR < 0.05 with the s0 parameter set to 0.1). Association networks were constructed using ShinyGO (Ge et al., 2020). Darker nodes are more significantly enriched protein sets, and bigger nodes represent larger protein sets. Thicker edges represent more overlapped proteins. Clusters were manually defined and annotated based on inspection of node descriptions and based on Gene Ontology obtained from String databases.^1^ Mercator4,^2^ agriGO v2.0 (Tian et al., 2017), and DAVID bioinformatics resources (Huang et al., 2009) were used to identify representative functional networks for the proteomics. Principal component analysis (PCA) plots were obtained from MetaboAnalyst^3^ and heatmaps were drawn using Perseus software.

## Results

**Figure 1:**
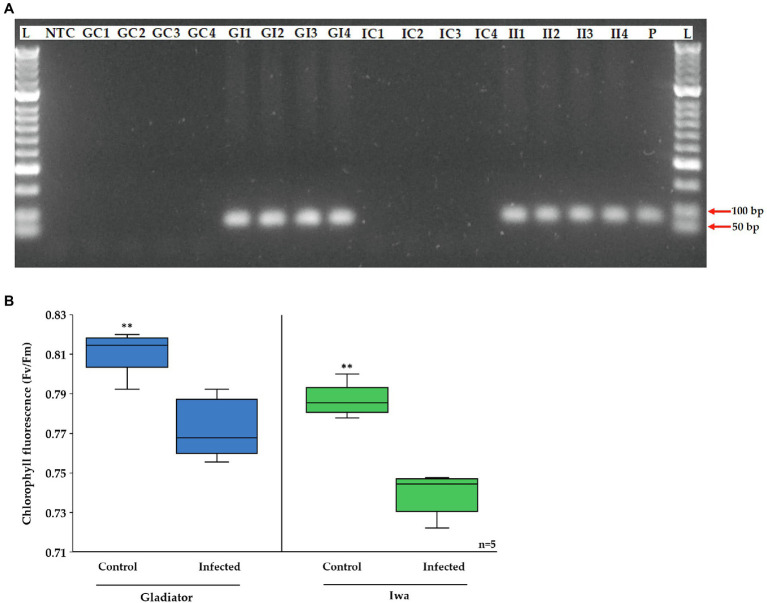
**(A)** Agarose gel electrophoresis of the PCR product of *Spongospora subterranean* 18S rRNA gene for DNA extracted from potato roots of inoculated and control plants. Lane M, MassRuler DNA ladder; lane NTC, non-template control; lane P, positive control; GC, Gladiator control; GI, Gladiator inoculated; IC, Iwa control; and II, Iwa inoculated. **(B)** Effect of potato root infection by *S. subterranean* on chlorophyll fluorescence. Values are means ± SE (*n* = 5 independent plants). Asterisk (**) indicate a significant difference at *p* < 0.01.

The aim of this study was to use a combined proteomics and phosphoproteomics approach to gain insight into the potato immune response to *S. subterranea* infection. We used two potato cultivars that differ in their susceptibility to *S. subterranea*. It is worth mentioning that while Gladiator and Iwa cultivars are classified as pathogen-resistant and susceptible, respectively, the inoculated plants of both cultivars formed root galls. However, the number of galls was fewer in Gladiator than Iwa (Supplementary Figure S1), which is consistent with previous greenhouse and field assessments of potato cultivars for resistance to *S. subterranea* (Falloon et al., 2016). Firstly, we confirmed that *S. subterranea* infection had occurred using PCR with primer pairs targeted to the 18S rRNA gene with an expected 95 bp product, using DNA extracted from the roots of inoculated and uninoculated plants (Figure 1A). The PCR results validated our samples with amplified *S. subterranea* 18S rRNA gene in all inoculated plants but not in any controls. We also measured the chlorophyll content in the infected and uninfected plants (Figure 1B) before collecting the samples for proteomics and phosphoproteomics analysis. Plant leaves can be an important tissue for practical monitoring of infection or resistance. An extensive change in the proteome profile of leaves in response to root infection by soilborne pathogen has been reported before (Coelho et al., 2021). Our results here showed that in both cultivars the amount of chlorophyll content significantly (*p* < 0.01) reduced after infection. However, the decrease in chlorophyll content after infection was more extensive in Iwa than in Gladiator.

### Overview of the Leaf Proteome of Potato Plants With Roots Inoculated With *Spongospora subterranea*

The proteome of potato leaves of the susceptible and resistant potato cultivars was analyzed using a shotgun quantitative proteomic approach. Our analysis identified nearly 2,700 proteins, of which several hundred proteins significantly (FDR < 0.05) changed in abundance across all experimental conditions (Table 1). The complete list of identified and significantly changed proteins is provided as Supplementary Table S1.

**Table 1:** Number of quantified and significantly changing proteins (FDR ≤ 0.05) from inoculated and uninoculated potato plants.

		Differentially abundant proteins (FDR < 5%)			
Total proteins	Identified	GI vs. GC		II vs. IC	
	2,669	1,341	Increased 744	893	Increased 432
			Decreased 597		Decreased 461

**Figure 2:**
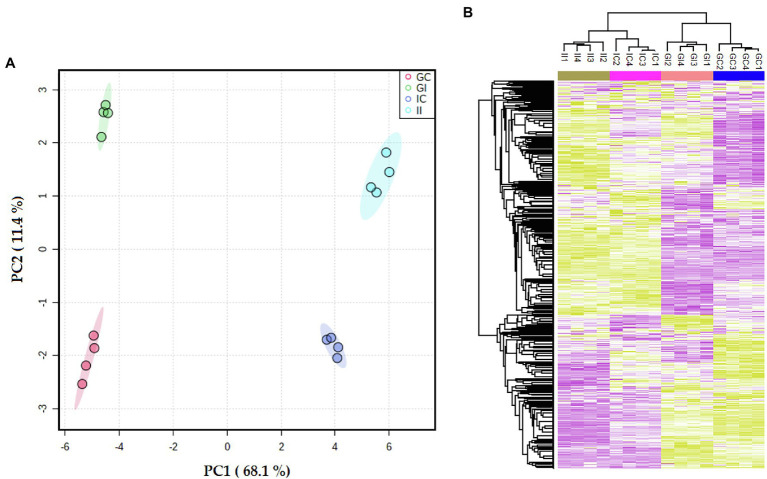
**(A)** Principal component analysis (PCA) differentiated the potato leaf proteomes of potato plants (Gladiator and Iwa) with roots non-inoculated and inoculated by *Spongospora subterranea*. **(B)** The Z-scored abundance of the complete set of identified proteins. GI, Gladiator infected; GC, Gladiator control; II, Iwa infected; and IC, Iwa control.

According to the PCA plot, the cultivar differences accounted for the largest observed variance in the potato leaf proteome (PC1 = 68%; Figure 2A). The second largest observed variance belonged to the difference between infected and control (uninfected) plants (PC2 = 11%). Accordingly, hierarchical clustering of the data and representation of Z-scored protein intensity values as a heat map also showed that sample replicates from each cultivar grouped together (Figure 2B).

**Figure 3:**
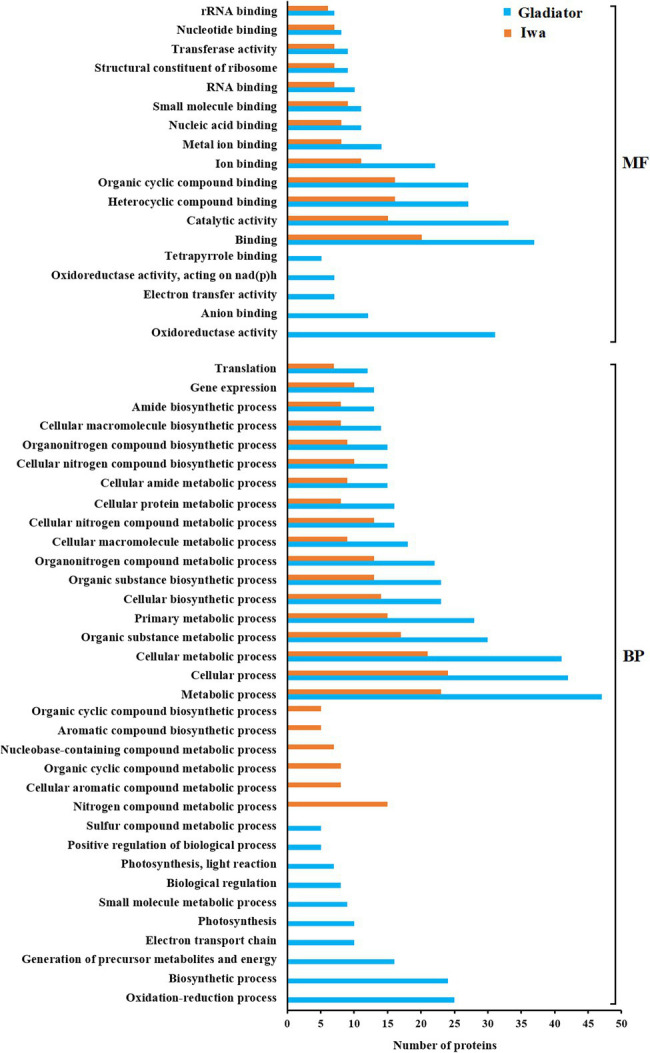
Gene ontology (GO) enrichment analysis of the differentially abundant leaf proteins of potato plants (Gladiator and Iwa) with roots non-inoculated and inoculated by *Spongospora subterranea*. MF, molecular function; BP, biological process.

Enrichment analysis of differentially abundant proteins (DAPs; infected vs. control) according to their molecular function (MF) and biological process (BP) ontological terms are shown in Figure 3. The most common MF categories associated with pathogen infection in both Gladiator and Iwa were associated with binding, catalytic activity, and transferase activity. Oxidoreductase activity, anion binding, electron transfer activity, and tetrapyrrole binding were MF categories that were specifically found in the resistant cultivar Gladiator but not in the susceptible cultivar Iwa (Figure 3 top). Metabolic process, cellular process, cellular biosynthetic process, gene expression, and translation were the most common BP categories in both Gladiator and Iwa. Oxidation–reduction process, biosynthetic process, generation of precursor metabolites and energy, electron transport chain, photosynthesis, small molecule metabolic process, and biological regulation were BP categories found in DAPs in Gladiator but not in Iwa. In contrast, nitrogen compound metabolic process, cellular aromatic compound metabolic process, organic cyclic compound metabolic process, nucleobase-containing compound metabolic process, aromatic compound biosynthetic process, and organic cyclic compound biosynthetic process were BP classes observed in Iwa only (Figure 3 bottom).

**Figure 4:**
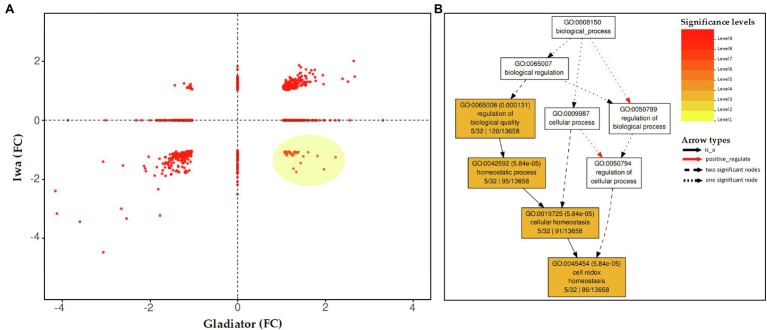
**(A)** Distribution of the differentially abundant leaf proteins (inoculated vs. non-inoculated) common to both potato cultivars (Gladiator and Iwa). The changes in proteins were presented as fold changes (FC). Distribution of the differentially abundant proteins (DAPs) in common between Gladiator and Iwa. The changes in proteins were presented as fold changes. **(B)** Enrichment analysis (BP) for the proteins that increased in leaves of cultivar Gladiator but decreased in cultivar Iwa 6 weeks post-inoculation.

To further interrogate the proteomic data set at the level of specific proteins, the subsets of proteins that were significantly altered upon *S. subterranea* infection in either one cultivar or both, DAPs were plotted according to their log_2_ fold change (infection vs. control). This two-way plot (Figure 4A) showed that 264 proteins were significantly increased in both cultivars, while 281 proteins were significantly decreased in both cultivars (Supplementary Table S2). To identify the key subset of proteins that might play a role in the resistance or susceptibility of potato to *S. subterranea*, we searched for those proteins that differentially accumulated between the two cultivars after infection. The highlighted area shows 36 proteins that were significantly (FDR < 5%) increased in the resistant cultivar but decreased in the susceptible cultivar (Supplementary Table S2). Furthermore, functional enrichment analysis (biological process) showed that most of these proteins were related to cell redox homeostasis (Figure 4B). We also found 15 proteins that increased in Iwa but decreased in Gladiator. However, no significant GO term was associated with this subset of proteins (Supplementary Table S2).

### Phosphoproteome of Potato Leaves in Response to Root Infection

**Figure 5:**
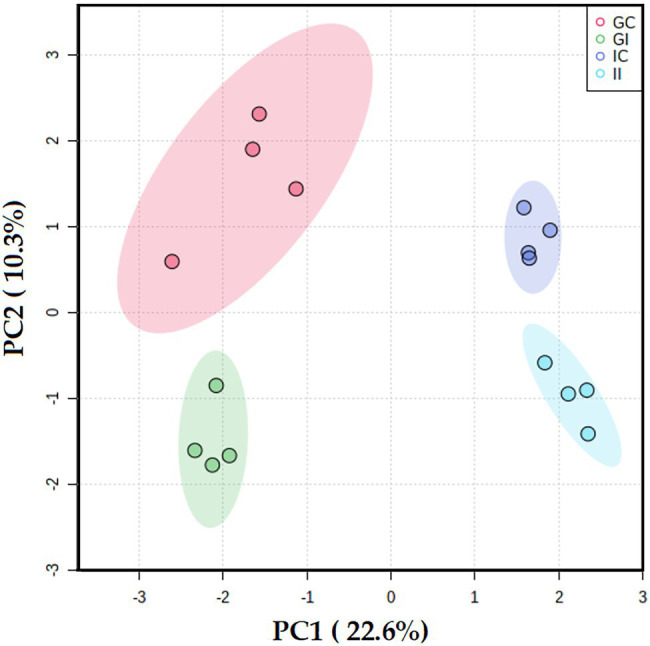
Principal component analysis differentiated the potato leaf phosphoproteomes of potato plants (Gladiator and Iwa) with roots non-inoculated and inoculated by *Spongospora subterranea*. GI, Gladiator infected; GC, Gladiator control; II, Iwa infected; and IC, Iwa control.

To investigate responses of potato to *S. subterranea* infection at the post-translational level, an MS-based approach was used to analyze the phosphoproteome of susceptible and resistant cultivars. Our analysis identified 2,640 phosphosites which were assigned to 1,498 phosphoproteins, as some proteins carried multiple phosphorylation sites. The complete list of identified phosphoproteins is provided as Supplementary Table S3. Furthermore, the PCA analysis revealed that the cultivars and the differences between infected and control (uninfected) plants were the primary sources of variance in phosphoproteomes of potato leaves (Figure 5).

**Figure 6:**
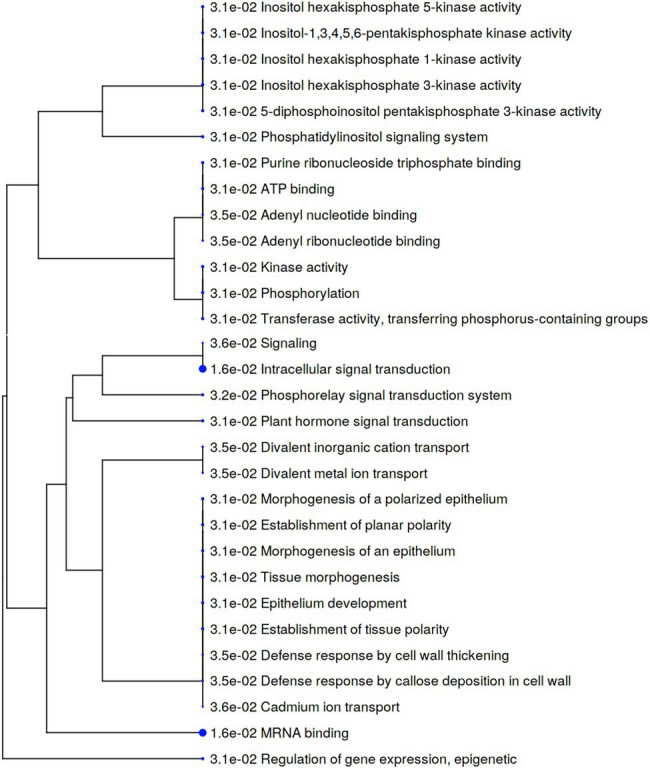
A hierarchical clustering tree summarizing the correlation among significant pathways of the increased differently abundant phosphoproteins in Gladiator (value of *p* < 0.05, log_2_ fold change > 2). Pathways with several shared genes are clustered together. Bigger dots (blue dots) indicate more significant *p*-values.

To identify phosphosites significantly affected by *S. subterranea* infection, we applied a *t*-test using *p*-value threshold of 0.05. In Gladiator, changes in abundance of 368 phosphosites were statistically significant, of which 229 were increased, and 139 were decreased in abundance after infection (Supplementary Table S3). In Iwa, 590 phosphosites significantly changed, of which 435 were increased and 155 were decreased (Supplementary Table S3). For the enrichment analysis, a filter was applied to include only those significantly changed phosphoproteins with log_2_ fold change above 2. We next used the increased differentially abundant phosphoproteins (value of *p* < 0.05, log_2_ fold change > 2) in Gladiator (GI vs. GC) or Iwa (II vs. IC) to identify cellular processes and metabolic pathways that might be involved in resistance against *S. subterranea*. The major effect of infection by *S. subterranea* at the phosphoproteome level, according to the phosphopeptides that were significantly increased in Gladiator, was on signal transduction and defense response (Figure 6). Specifically, GO terms that were related to signaling and defense in Gladiator included plant hormone signal transduction, phosphorelay signal transduction system, defense response by cell wall thickening, and defense response by callose deposition in cell wall (Figure 6).

**Figure 7:**
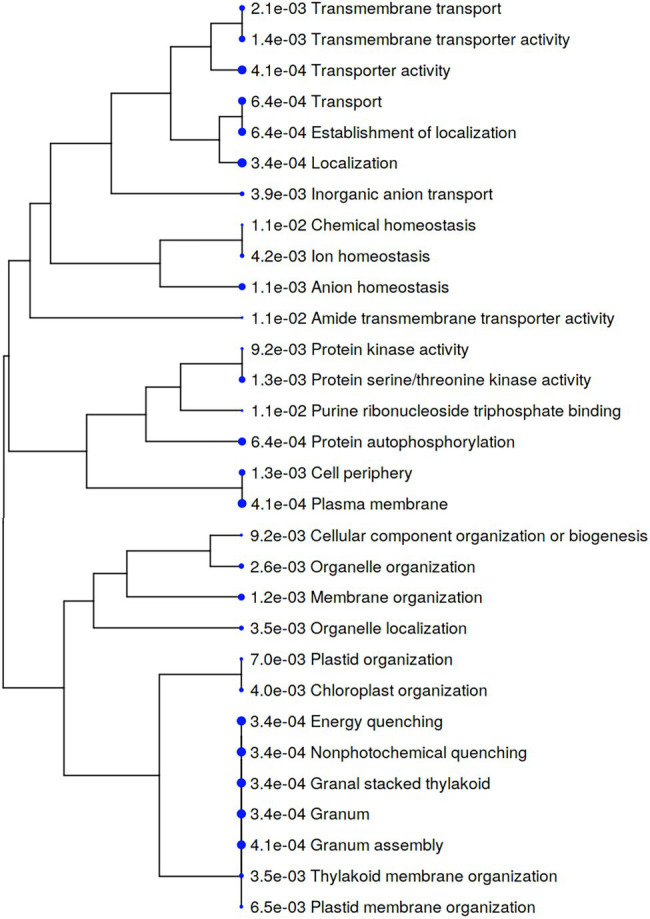
A hierarchical clustering tree summarizing the correlation among significant pathways of the increased differently abundant phosphoproteins in Iwa (value of *p* < 0.05, log_2_ fold change > 2). Pathways with several shared genes are clustered together. Bigger dots (blue dots) indicate more significant *p*-values.

In contrast, the majority of cellular processes represented by phosphoproteins that were significantly increased in Iwa after infection were related to transporter activity and localization (Figure 7). In particular, biological processes and molecular functions related to transportation including inorganic anion transport, transporter activity, transmembrane transporter activity, transmembrane transport, transport, localization, and establishment of localization were increased in Iwa after infection by *S. subterranea* (Figure 7).

## Discussion

In this study, we used large-scale proteomics and phosphoproteomics to analyze potato defense responses to *S. subterranea*. We identified several proteins and phosphoproteins that differentially changed between resistant and susceptible cultivars, highlighting the complexity of the potato defense regulatory system and *S. subterranea* disease processes. The present data provide new evidence that both cell redox homeostasis and signal transduction are strongly associated with potato resistance to *S. subterranea*.

In our proteomics analysis, we identified oxidoreductase activity, photosynthesis, and electron transport as key molecular functions and biological processes associated with the DAPs in Gladiator but not in Iwa (Figure 3). Functional annotation of those proteins that increased in Gladiator but decreased in Iwa was also obtained (Figure 4B). Cell redox homeostasis was the most significant biological process related to these proteins. The changes in the abundance of the proteins involved in the above processes have been detected in plant response to various biotic and abiotic stresses (Kundu et al., 2018; Amaral et al., 2021). Due to the metabolic variations caused by stress, the photosynthetic electron transfer chain and the cell redox potential may undergo massive changes and a decline in photosynthetic rate after pathogen invasion is well documented (Schenk et al., 2000; Macedo et al., 2003; Major and Constabel, 2006; Bozsó et al., 2009). Consistent with these findings, root infection by *S. subterranea* reduced the efficiency of primary conversion of light energy of PSII (chlorophyll fluorescence Fv/Fm value) in the leaves of both cultivars.

We noticed that the chlorophyll level was higher in Gladiator than Iwa in control plants, while after infection, the chlorophyll content in Gladiator was less affected in comparison with Iwa (Figure 1B). The ferredoxin–thioredoxin system activates light-driven metabolic reactions in plant leaves. We compared the abundance levels of ferredoxin and thioredoxin in the DAPs in both cultivars (Supplementary Figure S2). This analysis demonstrated that the abundance of these proteins was higher in both infected and uninfected Gladiator plants compared with Iwa. These results also confirmed that after infection, the abundance of most of these proteins increased in Gladiator but not in Iwa. Thioredoxins became reduced *via* the ferredoxin–thioredoxin reductase and PSI and provide a link between the activation of key photosynthetic enzymes and the activity of photosynthetic light reactions (Buchanan and Balmer, 2005). In photosynthesis, ferredoxin accepts electrons from PSI and reduces NADP^+^ *via* ferredoxin NADPH oxidoreductase. Ferredoxin also is involved in other reactions in the chloroplast, including redox regulation, amino acid synthesis, and sulfur and nitrogen assimilation. It was shown that the upregulation of ferredoxin reflects its direct participation in pathogen defense (Kuźniak and Kopczewski, 2020). The overexpression of ferredoxin in tobacco plants resulted in resistance to *Pseudomonas syringae* and *Erwinia carotovora* (Huang et al., 2007). Functional replacement of ferredoxin by a bacterial flavodoxin conferred resistance to the necrotrophic fungus *Botrytis cinerea* in tobacco (Rossi et al., 2017), and the infected plants showed a decreased ROS accumulation and a sustained photosynthetic electron flow. Wang et al. (2018b) showed that knockout of ferredoxin gene Fd2 (Fd2-KO) increased the plant’s susceptibility to *Golovinomyces cichoracearum*. This result confirmed the defects in the accumulation of ROS in the infected plants. Similar to ferredoxin, the involvement of thioredoxins in plant–microbe interactions has been investigated in several studies (Viefhues et al., 2014; Mukaihara et al., 2016; Nogueira-Lopez et al., 2018). In line with the above studies, our total protein analysis of leaves of the susceptible and resistant to root infection by *S. subterranea* confirmed the key role of electron transport (Figure 3), cell redox homeostasis (Figure 4), and ferredoxin–thioredoxin oxidoreductase (Supplementary Figure S2) in the potato resistance to this pathogen.

In our total protein analysis, we identified several defense-related proteins and antioxidant enzymes, including cinnamoyl-CoA reductase (CCR), ascorbate peroxidase1 (APX01), and chaperonin (TCPb and TCPe) that increased in the resistant cultivar but not in the susceptible cultivar (Supplementary Table S1). Cinnamoyl-CoA reductase is involved in lignin biosynthesis and plays an important role in the plant immune response (Park et al., 2017). The activation of CCR leads to the production of monolignols, increased ROS accumulation, and deposition of lignin. Thus, an increase in the activity of CCR will further activate plant defense responses against pathogens (Sun et al., 2019). Ascorbate peroxidase1 is an important antioxidant enzyme in plants. A decrease in the activity of APX upon biotic and abiotic stresses has been reported in the proteomics analysis of several other plant species (Margaria et al., 2013; Han et al., 2014). The accumulation of APX protein in Gladiator, therefore, has the capacity to increase resistance of this cultivar to *S. subterranea*. We found two different chaperonin proteins (chaperonin containing t-complex protein1 and beta subunit) that were increased in Gladiator but not in Iwa (Supplementary Table S1). These molecular chaperones are known to be involved in stabilization and assembly of actin and tubulin molecules. We found that the abundance of one of the tubulin proteins (accession number: M0ZYR0) was increased in Gladiator but not in Iwa (Supplementary Table S1). Molecular chaperones are involved in regulatory roles to prevent stress injury and the plant’s immune response (Ishikawa et al., 2003). In line with our results, the 60 kDa chaperonin protein was significantly increased in the resistant cultivar compared with susceptible cultivar in tomato plants after infection by *Pseudomonas solanacearum* (Afroz et al., 2009). Therefore, chaperonin proteins might contribute to the resistance of potato against pathogen attack.

Protein phosphorylation is one of the most common post-translational modifications, and it is known to regulate many plant molecular functions, including stress responses, signaling, and metabolism (Damaris and Yang, 2021). Our phosphoproteomics analysis of potato cultivars showed that the susceptible and resistant cultivar differentially responded to *S. subterranea* infection (Figure 5). In the resistant cultivar, several signaling, and defense-related processes were increased after infection in the phosphoproteome level (Figure 6). In the susceptible cultivar, however, the majority of increased processes belonged to transporter activity (Figure 7). Previous studies showed that protein phosphorylation plays a key role in regulating pattern-triggered immunity (PTI) signaling in plants and provided evidence that protein kinases participate in downstream signaling to induce defense responses in plants (Martin, 1999; Lin et al., 2014; Macho et al., 2014). Additionally, mitogen-activated protein kinases (MAPKs) are activated during plant defense responses by upstream MAPK kinases (MAPKKs; Colcombet and Hirt, 2008). In fact, the activation of MAPKKs and calcium-dependent protein kinases participate in downstream signaling to activate plant defense responses (Tena et al., 2011; Li et al., 2014). Through the phosphorylation/dephosphorylation of kinase cascade, the signals of pathogen infection stimuli are transmitted to the nucleus and activate the phosphorylation/dephosphorylation processes of disease resistant-related proteins to initiate the immune response (Li et al., 2022). Consist with this, we found several functions related to kinase activity and phosphorylation that increased after infection in Gladiator but not in Iwa (Figures 6, 7). Our phosphoproteome analysis also confirmed the decrease in abundance of pyruvate phosphate dikinase (PPDK) in the susceptible cultivar (Supplementary Table S3). PPDK belongs to the family of transferases and plays a crucial role in plant resistance against pathogens (Wang et al., 2012; Lu et al., 2019). The induction of PPDK in abiotic stresses including cold, salt stress, low oxygen, heavy metals, and osmotic stress (Moons et al., 1998; Dong et al., 2016; Wang et al., 2018a), and biotic stress, such as viral infection (Spoustová et al., 2015; Lu et al., 2019), have been reported before. Thus, the decrease in the abundance of PPDK in Iwa might reduce the resistance to *S. subterranea* infection.

In conclusion, our proteomics and phosphoproteomics analysis of potato cultivars in response to *S. subterranea* infection provide a better understanding of the regulatory mechanisms that are involved in resistance response to the pathogen. We identified specific effects on redox homeostasis, electron transport, carbohydrate metabolism, and kinase activity in the resistant cultivar Gladiator upon *S. subterranea* infection. These results extend the current knowledge of *Spongospora*-infected potato and its resistance responses from the perspective of combined proteomics and phosphoproteomics analysis.

## Data Availability Statement

The datasets presented in this study can be found in online repositories. The names of the repository/repositories and accession number(s) can be found at: the datasets generated for this study can be found in the ProteomeXchange Consortium *via* the PRIDE (Perez-Riverol et al., 2019) partner repository with the dataset identifier PXD031529.

## Author Contributions

SB, RW, CW, RT, and DN designed the study. SB and RW developed the methodology and analyzed the data and performed the bioinformatics analysis. SB performed the experiments and wrote the manuscript. RW acquired the proteomics data. CW, RW, RT, and DN reviewed and edited the manuscript. CW and DN financially supported the project. All authors contributed to the article and approved the submitted version.

## Funding

This research was funded by the Australian Research Council, Discovery Grant program, DP180103337.

## Conflict of Interest

The authors declare that the research was conducted in the absence of any commercial or financial relationships that could be construed as a potential conflict of interest.

## Publisher’s Note

All claims expressed in this article are solely those of the authors and do not necessarily represent those of their affiliated organizations, or those of the publisher, the editors and the reviewers. Any product that may be evaluated in this article, or claim that may be made by its manufacturer, is not guaranteed or endorsed by the publisher.

## Footnotes

^1^ https://string-db.org/

^2^ https://plabipd.de/portal/mercator4

^3^ https://www.metaboanalyst.ca

## Supplementary Material

**Supplementary Table S1** Complete list of identified and significantly changed proteins.

**Supplementary Table S2** Subset of proteins that increased in Gladiator and decreased in Iwa.

**Supplementary Table S3** Complete list of identified and significantly changed phosphoproteins.

**Supplementary Data Sheet 1** Supplementary data legends and figures.
