# Supplementary material for: Large-Scale Protein and Phosphoprotein Profiling to Explore Potato Resistance Mechanisms to Spongospora subterranea Infection
Source: Front Plant Sci. 2022 Apr 14;13:872901. doi: 10.3389/fpls.2022.872901 (PMC9047998; doi:10.3389/fpls.2022.872901)
Supplement: Supplementary file 4 [file Data_Sheet_1.docx]

Supplementary Material

Supplementary data legends:

Document S1: Supplementary data legends and Figures

Table S1: Complete list of identified and significantly changed proteins

Table S2: List of total protein that increased in Gladiator and decreased in Iwa

Table S3: Complete list of identified and significantly changed phosphoproteins

## Supplementary Figures S1.

**
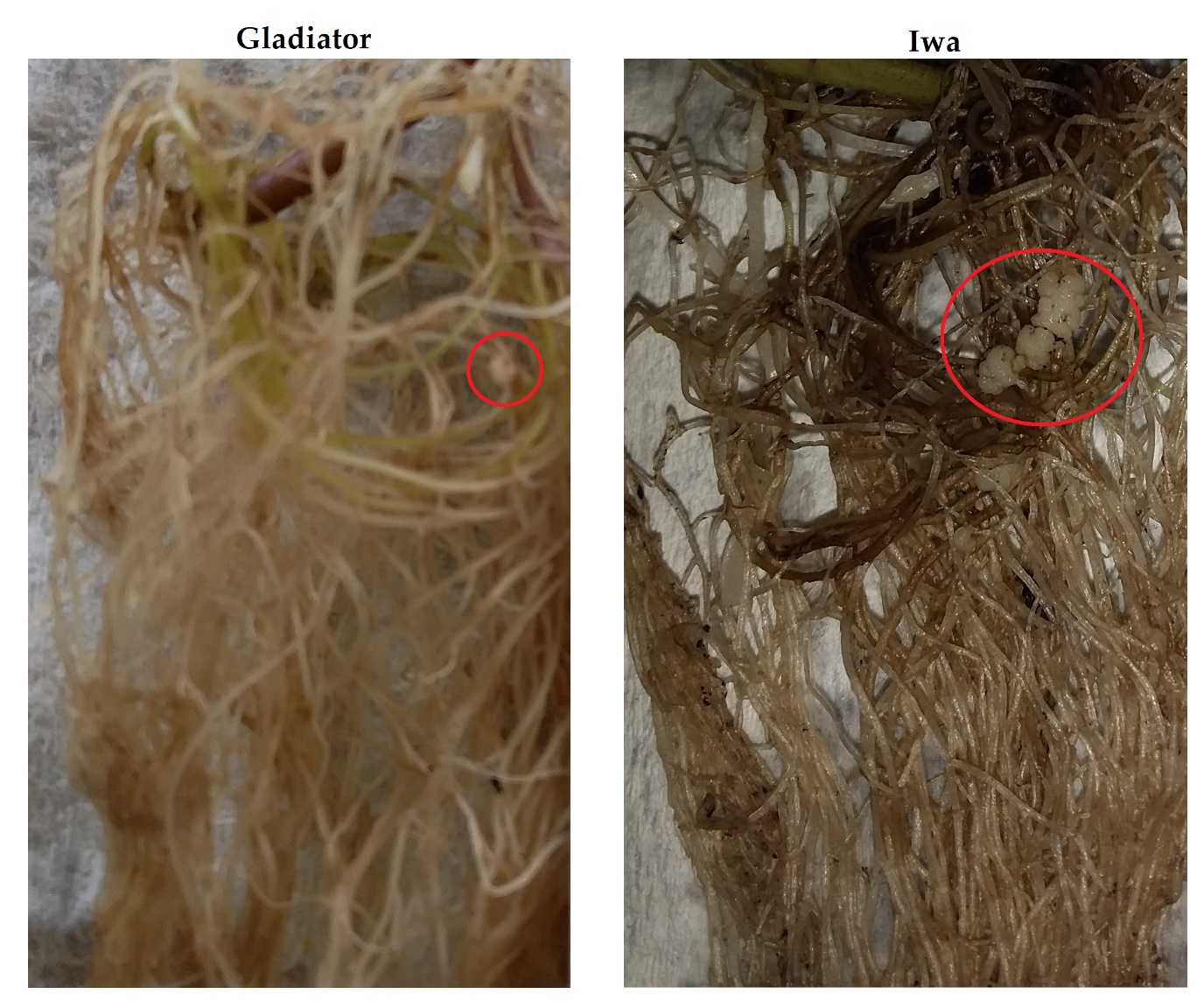
**

**Supplementary Figure 1.** Root gall formation in the inoculated potato cultivars. Gladiator (left) and Iwa (right). Both cultivars developed root galls 42 days after infection (indicated with red circle in picture). However, the number of galls as well as their size was bigger in Iwa.

**Supplementary Figure S2.**


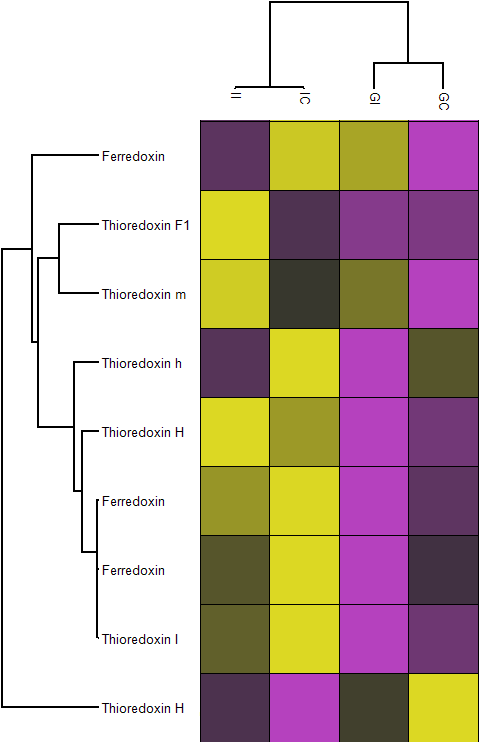


**Supplementary Figure 2.** The Z-scored abundance of the ferredoxin and thioredoxin proteins in the differentially abundant proteins (DAPs) in Gladiator and Iwa. GI: Gladiator infected; GC: Gladiator control; II: Iwa infected; IC: Iwa control.
